# Quantifying mechanical force in axonal growth and guidance

**DOI:** 10.3389/fncel.2015.00359

**Published:** 2015-09-16

**Authors:** Ahmad I. M. Athamneh, Daniel M. Suter

**Affiliations:** Bindley Bioscience Center, Birck Nanotechnology Center, Department of Biological Sciences, Purdue UniversityWest Lafayette, IN, USA

**Keywords:** growth cone biomechanics, axon elongation, mechanotransduction, cytoskeleton, biophysics, traction force

## Abstract

Mechanical force plays a fundamental role in neuronal development, physiology, and regeneration. In particular, research has shown that force is involved in growth cone-mediated axonal growth and guidance as well as stretch-induced elongation when an organism increases in size after forming initial synaptic connections. However, much of the details about the exact role of force in these fundamental processes remain unknown. In this review, we highlight: (1) standing questions concerning the role of mechanical force in axonal growth and guidance; and (2) different experimental techniques used to quantify forces in axons and growth cones. We believe that satisfying answers to these questions will require quantitative information about the relationship between elongation, forces, cytoskeletal dynamics, axonal transport, signaling, substrate adhesion, and stiffness contributing to directional growth advance. Furthermore, we address why a wide range of force values have been reported in the literature, and what these values mean in the context of neuronal mechanics. We hope that this review will provide a guide for those interested in studying the role of force in development and regeneration of neuronal networks.

## Introduction

The role mechanical forces play in the development and maintenance of neuronal networks has been increasingly recognized and addressed (Bray, 1979; Lamoureux et al., 1989; Suter and Miller, 2011; Franze et al., 2013). Many aspects of axonal growth and development have been examined in the context of mechanical force including cytoskeletal dynamics (Lee and Suter, 2008; Schaefer et al., 2008), axonal transport (Loverde et al., 2011; O’Toole and Miller, 2011; Ahmed and Saif, 2014), growth cone guidance (Suter et al., 1998; Suter and Forscher, 2001; Moore et al., 2009), and molecular motor activity (Bridgman et al., 2001). It has been shown that growth cones generate traction force (Lamoureux et al., 1989; Heidemann et al., 1990) and respond to mechanical stress (Franze et al., 2009) or change in substrate rigidity (Chan and Odde, 2008; Koch et al., 2012). Furthermore, it has been found that mechanical tension induces axonal growth (Bray, 1984; Zheng et al., 1991; Pfister et al., 2004) and that axonal tension is tightly regulated (Lamoureux et al., 1989; Rajagopalan et al., 2010; Hyland et al., 2014). Despite significant advances, many aspects of the mechanical control of axonal growth and guidance as well as maintenance of axons after synapse formation remain unclear.

## Standing Questions Pertaining to the Role of Force in Neuronal Processes

**Figure 1:**
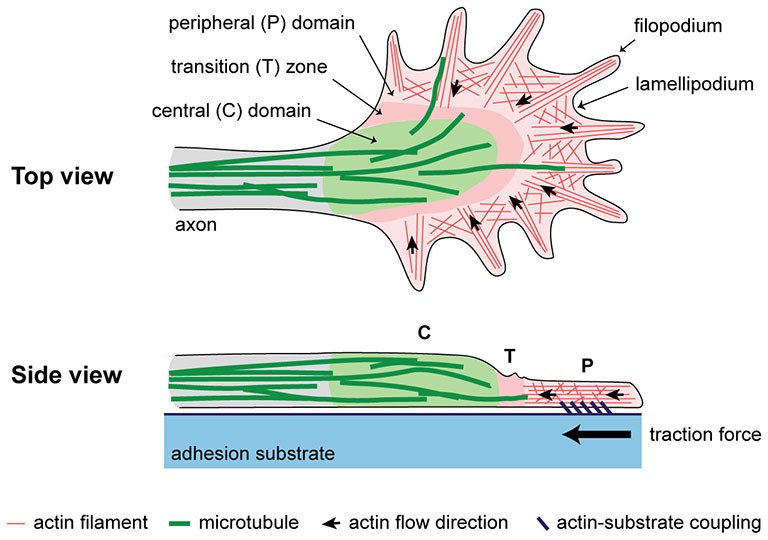
**A simplified schematic of the cytoplasmic domains and cytoskeletal structures in the growth cone**. Traction force is generated as a result of coupling of F-actin flow in the P domain to an extracellular adhesion substrate.

There is ample evidence that axonal elongation is influenced by both biochemical and biomechanical factors. The neuronal growth cone controls the direction and rate of axonal growth by navigating the surrounding environment searching for molecular, mechanical, and topographical cues. The machinery responsible for sensing stiffness of the extracellular matrix (ECM) as well as of neighboring cellular surfaces is primarily powered by the actin cytoskeleton, which is highly dynamic and is constantly turning over in the peripheral (P) domain and transition (T) zone (Figure 1). Actin polymerizes at the leading edge and is pulled backward by myosin motors, resulting in retrograde F-actin flow (Lin et al., 1996; Medeiros et al., 2006). Traction force is generated as a result of coupling of the F-actin flow to cellular and ECM substrates through adhesion receptors, such as immunoglobulin superfamily cell adhesion molecules, N-cadherin, and integrins (Suter et al., 1998; Bard et al., 2008; Shimada et al., 2008). Several reviews have discussed this substrate-cytoskeletal coupling model including what is known about the role of the cytoskeleton, molecular motors as well as signaling pathways involved (Suter and Forscher, 2000; Suter and Miller, 2011; Gomez and Letourneau, 2014).

## How Does Substrate Stiffness Affect Axonal Growth?

While it has been shown that the neuronal growth cone controls the advance of axons in part by sensing and responding to the substrate stiffness, little is known about the physical parameters governing these mechanisms. According to the prevailing model, the level of traction force is determined by the abundance and strength of coupling between F-actin flow and cellular surfaces or ECM. Coupling is influenced by the presence of adhesion substrates as well as the stiffness of the environment. The substrate must be compliant enough to allow adhesion to mature and form strong coupling capable of bearing increasing tension. If the substrate is too stiff, newly formed adhesions abruptly break as lack of compliance in the substrate results in rapid building of tension. This “motor-clutch” model provides a mechanism that inherently senses and responds to substrate stiffness (Chan and Odde, 2008). Consistent with this model, experiments have demonstrated the effect of substrate stiffness on coupling and traction force development in growth cones, but the consequences on neurite outgrowth are not fully clear yet. Furthermore, different types of neuron responded differently to substrate stiffness. For example, softer substrates were shown to promote better neurite growth and branching in spinal cord (Flanagan et al., 2002; Jiang et al., 2008) and hippocampal neurons (Kostic et al., 2007), but not cortical neurons (Norman and Aranda-Espinoza, 2010). Another study found that stiffer substrates significantly increased neurite outgrowth in cortical neurons (Stabenfeldt and LaPlaca, 2011). In a later study, neurite outgrowth of dorsal root ganglion neurons from the peripheral nervous system (PNS) were sensitive to changes in substrate stiffness unlike hippocampal neurons from the central nervous system (CNS; Koch et al., 2012), which seems to contradict earlier observations (Franze et al., 2013). It is important to note that the range of substrate stiffness employed in these studies are not all the same, which makes a direct comparison of the results more challenging, even for the same type of neuron. The apparent discrepancies in the literature, which indicate that different neurons may exhibit different mechanosensitivity, have if anything kept alive the debate about the effect of substrate stiffness on axonal growth, and certainly challenged our understanding of the mechanisms underlying mechanotransduction in neuronal growth cones.

Recently, it has been shown that compliance, necessary to allow time for coupling to mature, is provided by elastic micron-scale deformations in the actomyosin network and the nascent adhesions themselves (Mejean et al., 2013). Adding to the complexity is the observed biphasic dependence of traction force on the substrate stiffness; i.e., traction force generated by nonneuronal and neuronal cell has been shown to increase linearly with the substrate stiffness but then plateau at sufficiently higher substrate stiffness (Saez et al., 2005; Ghibaudo et al., 2008; Koch et al., 2012; Trichet et al., 2012; Yip et al., 2013). This biphasic behavior complicates comparative analysis amongst studies using different experimental techniques or substrates of different stiffness.

## Do Growth Cones Respond to Mechanical Force or Deformation?

The lack of understanding of the mechanisms underlying mechanotransduction is manifested in the ongoing debate whether cell respond to force (stress) or deformation (strain) (Saez et al., 2005; Yip et al., 2013). In our lab, we have shown that the absolute level of substrate deformation appears to be a better predictor of adhesion-mediated growth cone advance when compared to the level of traction force (Athamneh et al., in press). We probed *Aplysia* growth cones with microneedles (MNs) having stiffness ranging from 0.003 to 0.1 N/m, and found that the threshold deformation for a positive growth cone response was about 1 μm of MN deflection regardless of how much force was exerted by the growth cone (Athamneh et al., in press). Therefore, our data highlight the fundamental role of strain vs. stress in controlling the mechanical response during cell migration. Other studies on mechanosensing have also shown evidence of micron-scale deformations involved in sensing and responding to substrate stiffness. For example, rat embryo fibroblasts cultured on a micropillar array caused a constant deformation of 0.84 μm regardless of micropillar stiffness (Trichet et al., 2012). Furthermore, it was found that the mechanical properties of substrate-cytoskeleton linkages in *Aplysia* growth cones were dominated by elastic structures that undergo micron-sized reversible deformations (Mejean et al., 2013). It is not entirely clear at this time what is the physiological significance of the micron-scale deformation, or how can it be reconciled with our understanding of mechanotransduction. However, these micron-scale deformations imply that mechanotransduction not only involves cell adhesion receptors and coupling proteins (Suter et al., 1998; Bard et al., 2008; Shimada et al., 2008), stretch-induced phosphorylation (Suter and Forscher, 2001; Kostic et al., 2007) and activation of ionic channels (Rajnicek and McCaig, 1997; Gomez et al., 2001; Franze et al., 2009; Kerstein et al., 2013), but also involves cytoskeletal reorganization spanning a significant micron-scale spatial range. Clearly, more work is needed to understand the relationship between events taking place at the nano-scale molecular level and cytoskeletal and cytoplasmic domain rearrangements at the micron-scale level in addition to the signaling/feedback mechanisms spanning the two length scales.

## How Does the Growth Cone Build Up Traction Force in Substrate-Mediated Growth?

Related to feedback signaling transcending multiple length scales is the question: how does the growth cone build up traction force in substrate-mediated growth? We have shown that traction force increases gradually over time as the growth cone encounters a new adhesion substrate. The maximum level of the force generated depends on the stiffness of the new substrate, implying continuous strengthening of the clutch and/or active recruitment of molecular motors (Athamneh et al., in press). Similar results were reported for rat embryo fibroblasts cultured on a micropillar array (Trichet et al., 2012). We also know from previous studies that the growth cone response to a physically restrained adhesion substrate includes adhesion formation, Src tyrosine phosphorylation, slowing of retrograde F-actin flow, increased actin assembly, advancing of microtubules, and leading edge advance (Suter et al., 1998, 2004; Suter and Forscher, 2001; Lee and Suter, 2008; Schaefer et al., 2008). Despite all this knowledge, the details of how the different events are orchestrated to gradually build up traction force, up to 10^2^ nN range (Athamneh et al., in press), and guide axonal growth in the direction of the adhesion site are not entirely clear.

## What is the Actual Role of Molecular Motors in Growth Cone Steering?

Experimental evidence suggests that in addition to actin and microtubule assembly dynamics, a number of molecular motors are involved in adhesion-mediated growth cone steering. Myosin II is essential for generating retrograde actin flow (Lin et al., 1996; Bridgman et al., 2001), growth cone steering (Turney and Bridgman, 2005), and actin-filament recycling in the T zone (Medeiros et al., 2006). Dynein is important for microtubule forward movement during growth cone steering and axonal elongation (Myers et al., 2006; Grabham et al., 2007; Roossien et al., 2014). Additionally, it has been suggested that kinesin-5 is involved in growth cone steering by opposing the action of cytoplasmic dynein to affect selective polarization of microtubules in the P domain (Nadar et al., 2008). Furthermore, kinesin-1-driven microtubule sliding promotes initial axonal extension in developing *Drosophila* neurons (Lu et al., 2013). However, the details of motor activity during adhesion-mediated growth, including what structures support these motor activities in order to generate pushing/pulling forces, are not known. Also not clear is how motor activity in growth cones is influenced by different substrates (Lin et al., 1996; Turney and Bridgman, 2005; Medeiros et al., 2006; Ketschek et al., 2007; Rösner et al., 2007; Kollins et al., 2009).

## What is the Role of Mechanical Force in Regulating Axonal Transport?

On the axon level, it has been show that tension is tightly regulated (Lamoureux et al., 1989; Rajagopalan et al., 2010; Hyland et al., 2014) and induces axonal growth (Bray, 1984; Zheng et al., 1991; Chada et al., 1997; Pfister et al., 2004), although the mechanism is not yet fully understood (Suter and Miller, 2011). For axons to grow, components must be transported. It follows that understanding the effect of tension on axonal transport is key to understanding the mechanisms of tension-induced growth, although very little is known in this area (O’Toole and Miller, 2011). Increased tension has been shown to decrease fast mitochondria transport in DRG neurons (Loverde et al., 2011), but increased fast vesicles transport in *Aplysia* neurons (Ahmed and Saif, 2014). It was suggested that tension also influences motor activity in the axon, although the details of what motors are affected by tension are not known (Ahmed and Saif, 2014).

In summary, many questions related to the role of force in axonal growth and guidance remain unanswered. We believe that satisfying answers to these questions will require quantitative information about the relationship between elongation, forces, cytoskeletal dynamics, axonal transport, signaling, substrate adhesion, and stiffness contributing to axonal growth and guidance. In the following section, we list experimental techniques used to quantify forces in axons and growth cones and discuss relevant findings.

## Techniques for Quantitative Force Measurements in Axons and Growth Cones

### Force-Calibrated Microneedles

Bray (1984) was the first to use MNs to demonstrate that neurites grow in response to mechanical tension. Earlier, Bray (1979) used vectorial analysis of the outlines of individually isolated sensory neurons to produce the first evidence that growth cones pull neurites by generating traction force. First quantitative force measurements in axons were performed using force-calibrated MNs by the Heidemann group (Dennerll et al., 1988). A permanent “rest tension” was identified in PC-12 neurites ranging over three orders of magnitude (10^−2^–10^0^ nN) (Dennerll et al., 1988). In these experiments, the MN was attached to the neurite at a middle point between the soma and the growth cone, and then moved rapidly perpendicular to the neurite’s long axis causing tension in the neurite and needle deflection. The same group later attached a MN to the soma of a cultured chick sensory neuron and raised it so that the cell became attached to the substrate at the growth cone only. Neurite tension was found to be in the 10^0^ nN range and strongly correlated with growth cone advance (Lamoureux et al., 1989). Later, the same group showed that axonal elongation correlated with applied tension using a MN (Zheng et al., 1991). Recently, O’Toole et al. (2015) developed a mathematical model to discern individual subcellular forces within the axon and growth cone by relating theses forces to the net axonal tension measured with a MN. Determination of subcellular forces was enabled by labeling of docked mitochondria to monitor subcellular strain. The mean force generated by the rear of the growth cone and axon was 2.0 and 0.6 nN, respectively, suggesting that contractile forces are generated in microtubule-rich regions at the rear of the growth cone and along the axon. We have used MNs in our laboratory to measure traction force in *Aplysia* growth cones as they respond to an adhesion substrate (Athamneh et al., in press).

MNs provide a simple, direct, and effective method for applying and measuring forces in neurons with sensitivity in the 10^−3^ nN range, although the technique can be time-consuming. It does not require specialized instruments or sophisticated analysis procedures. However, the technique is very sensitive to vibration and care must be taken not to cause damage to the cell. Additionally, whereas MNs were effective in measuring traction force in large *Aplysia* growth cones, they can be too large for measurements in other cell types with smaller growth cones. When cultured on poly-L-lysine, *Aplysia* growth cones on average cover an area of approximately 1.25 × 10^3^ μm^2^ (Wu et al., 2008), which is 5–10 times larger than growth cones from other species.

### Traction Force Microscopy

In traction force microscopy (TFM), cells are cultured on a deformable substrate, which can be a hydrogel or a nanowire array. Fluorescent beads are embedded as markers within the substrate to facilitate optical detection of the deformation caused by the cell. Force exerted by the cell is calculated using measured deformations and the stiffness of the substrate. Due to the popularity of TFM in the field of cell biomechanics, numerous reviews and method articles have been published describing the theory behind the techniques as well as methods for substrate preparation and fabrication (Sabass et al., 2008; Plotnikov et al., 2014; Style et al., 2014; Schwarz and Soiné, 2015). Bridgman et al. (2001) used the technique to study the role of Myosin IIB in generation of filopodia-mediated traction force in growth cones from mouse superior cervical ganglion neurons. The traction force generated by a single filopodium was found to be in the 10^0^ nN range and partially reduced in Myosin IIB knock-out neurons, suggesting that Myosin IIB was acting in combination with other myosins (Bridgman et al., 2001). Chan and Odde (2008) used TFM to validate their “motor-clutch” model. They identified a 1 kPa threshold for substrate stiffness below which a single filopodium of embryonic-chick—forebrain neurons exhibited oscillatory load-and-fail dynamics, with slower retrograde flow and higher traction forces. On substrates stiffer than 1 kPa, filopodia showed frictional slippage, with fast retrograde flow and low traction forces (Chan and Odde, 2008). Koch et al. (2012) compared traction forces generated by growth cones of rat dorsal root ganglion and hippocampal neurons and found that the growth cones of PNS neurons produce higher traction forces compared to CNS neurons (10^0^ vs. 10^−1^ nN range). For both neuron types, traction forced increased with increasing stiffness, which is consistent with other studies. Toriyama et al. (2013) observed that netrin-1 positively regulates traction force in the growth cone of rat hippocampal neurons through Pak1-mediated shootin1 phosphorylation, which enhances F-actin-substrate coupling leading to higher force generation. Traction force was found to be highest in the actin-rich P domain of *Aplysia* growth cones, and although traction force redistributes continuously, the net resulting neurite tension was tightly regulated around 3.1 nN (Hyland et al., 2014). In NG108-15 neuroblastoma cells neurite tension was measured as 0.6 nN (Betz et al., 2011), which is consistent with earlier neurite tension measurements in PC-12 cells using MNs (Dennerll et al., 1988). Finally, using a microfabricated nanowire array TFM revealed a wide range of traction forces (10^−2^–10^1^ nN) in the growth cones of rat dorsal root ganglion neurons depending on nanowire stiffness (Hällström et al., 2010).

While TFM provides high-resolution force measurements at the growth cone and single filopodia levels, the uniformity of the substrate stiffness does not mimic the complex environment *in vivo* particularly well. The introduction of a stiffness gradient or a defined stiffness micropattern for neurons to interact with remains a technical challenge. Accordingly, force values reported using conventional TFM on a uniform substrate may represent a “quasi” steady state condition and not traction force generation in response to a change in adhesion substrate.

### Optical Tweezers

The optical tweezer (OT; or optical trap) setup consists of a highly focused infrared laser beam that can physically hold (i.e., trap) a microbead. The tweezers can be calibrated to know how much force (typically in 10^−3^–10^0^ nN range) is required to remove the trapped bead from its focal center. Because of its high force sensitivity, this technique has been extensively used to measure molecular forces produced by proteins, especially molecular motors (Elting and Spudich, 2012). Using OTs, it was shown that the forces generated by the filopodia in the growth cones of CNS (hippocampal) neurons were larger when compared with PNS (Dorsal Root Ganglia) neurons (5 × 10^−3^ vs. 1–2 × 10^−3^ nN) (Amin et al., 2013). The lamellipodia of both CNS and PNS neurons generated similar lateral forces level up to 20 × 10^−3^ nN, but exerted larger vertical force in PNS neurons (4 × 10^−3^ vs. 1–5 × 10^−3^ nN) (Amin et al., 2013). Mejean et al. (2013) used an optical trap to characterize the mechanics of apCAM-mediated nascent adhesions in *Aplysia* growth cones and found that for forces in the 10^−3^ nN scale, nascent adhesions were dominated by an elastic structure, which can be reversibly deformed by up to 1 μm (Mejean et al., 2013). These results suggested a substrate-cytoskeleton interface dominated by a compliant cross-linked network and not a number of stiff molecular springs in parallel. The compliance of the network may provide more time for nascent adhesions to strengthen before larger forces develop. Also using an optical trap, Bard et al. (2008) studied N-cadherin-mediated substrate-cytoskeletal coupling and found that at low forces (<16 × 10^−3^ nN) slippage of cadherin-cytoskeleton bonds occurred, while at high forces actin accumulated strengthening nascent N-cadherin coupling. Moore et al. (2009) showed that the advancing growth cones of spinal commissural neurons generated traction force greater than 63 × 10^−3^ nN when confronted with a restrained netrin-1 bead (Moore et al., 2009). Without the bead, the same growth cone generated 9 × 10^−3^ nN local traction force as determined by TFM.

Using OTs for force measurements can be technically involved. However, once established, the technique can provide superb resolution and spatial selectivity. It also overcomes the issue of substrate uniformity suffered by TFM. A major limitation of OTs is that the level of force that can be applied without causing damage to the cells is in the 10^−3^ nN range. Thus, OTs typically cannot sustain enough force to induce adhesion-mediated growth cone advance or steering responses (Bard et al., 2008; Shahapure et al., 2010; Amin et al., 2013; Mejean et al., 2013), especially in neurons with large growth cones such as *Aplysia*.

### Atomic Force Microscopy

Atomic force microscopy (AFM) can be used both for applying and measuring forces in neurons (Franze et al., 2009), although it has been mostly used for imaging and measuring cell topography and elasticity (McNally and Borgens, 2004; Grzywa et al., 2006; Xiong et al., 2009; Spedden et al., 2012). Using AFM in the lateral force measurements mode, Fuhs et al. (2013) measured the forward pushing forces of mouse retinal ganglion cell and NG108-15 growth cones and found them to be on the order of 10^−1^ nN. In the same paper, the authors reported that the total traction force generated by the NG108-15 growth cones as measured by TFM was two orders of magnitude higher. While AFM lateral force measurements may appear to be an ideal solution for measuring traction force in growth cones, significant technical difficulties exist particularly related to calibrating the torsional response of the AFM cantilever (Karhu et al., 2009). Karhu et al. (2009) showed that frictional-force measurement using AFM was possible in longitudinal imaging mode and provided several advantages over lateral imaging mode. In our lab, we have developed a new AFM-based method for measuring retrograde traction force in growth cones that does not require lateral force calibration by following the approach developed by Karhu et al. (2009). We used the new method with an apCAM-coated colloidal cantilever to measure the temporal traction force profile in *Aplysia* growth cones as they encounter a physically-restrained adhesion substrate (Athamneh et al., in press). In summary, commercially available AFM systems provide high-resolution data over a large force range from 10^−3^ to 10^2^ nN. However, calibration and data analysis require significant involvement. Additionally, care must be taken to account for noise and instrumental drift, especially in temporal measurements for an extended period of time.

### MEMS Force Sensors

Microelectromechanical system (MEMS)-based force sensors are microfabricated from a single silicon crystal and can provide high-resolution quantitative measurements over a large dynamic range (Rajagopalan and Saif, 2011). Using a MEMS sensor, it was found that axons of embryonic *Drosophila* neurons that have formed neuromuscular junctions maintain a rest tension of 1–13 nN (Siechen et al., 2009; Rajagopalan et al., 2010), which is in good agreement with the *in vitro* studies determining neurite rest tension with either TFM (Betz et al., 2011; Hyland et al., 2014) or MNs (Dennerll et al., 1988). Axons responded to perturbation of the rest tension by either relaxing or contracting to restore original rest tension (Rajagopalan et al., 2010). The advantages and limitations of MEMS-based force sensors for the study of cell biomechanics have been reviewed by Rajagopalan and Saif (2011).

### Summary and Conclusions

**Figure 2:**
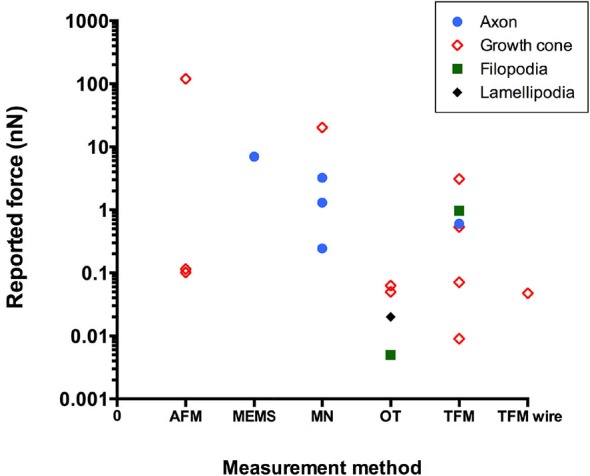
**Reported literature values of force in neurons using different experimental techniques (AFM, atomic force microscopy; MEMS, microelectromechanical system-based force sensors; MN, microneedle; OT, optical tweezers; TFM, traction force microscopy)**.

Figure 2 and Table 1 show force values reported in the literature for different parts of the neuron using different experimental techniques. The graph illustrates the wide range of force values even for the same experimental technique or the same part of the neuron. For example, values reported for traction force generated by the growth cone ranged over five orders of magnitude. Indeed, different force values can be expected for different types of neurons and different sizes of neuronal areas probed, which will result in different amounts of cytoskeletal structures and motors that are engaged in the process. Furthermore, different experimental techniques may provide slightly different values for the same neuron and area. However, it is possible that some of the variations in force values could be due to calibration problems. If anything, the discrepancy illustrated in Figure 2 suggests that extreme caution must be taken when comparing force values reported in different studies. Setting up standardized calibration methods for each force measurement method might be an approach to reduce some of the variations currently found in the literature.

**Table 1:** **Force measurements reported in the literature for different cell types and experimental techniques**.

Cell type	Part of the cell probed	Reported force value (nN)		
		Lowest	Highest	Mean	Method	Reference
PC-12	Axon	0	10	0.244	MN	Dennerll et al. (1988)
Embryonic chick sensory neurons	Axon	0.46	6		MN	Lamoureux et al. (1989)
Superior cervical ganglion neurons	Filopodia		1.6	0.97	TFM	Bridgman et al. (2001)
*Aplysia Californica* bag cell neurons	Growth cones	0.5	8.5	3.1	TFM	Hyland et al. (2014)
Rat dorsal root ganglion	Growth cones		1.179	0.537	TFM	Koch et al. (2012)
Rat central nervous systems neurons	Growth cones		0.247	0.071	TFM	Koch et al. (2012)
Rat dorsal root ganglion neurons	Growth cones	0.015	0.08		TFM wire	Hällström et al. (2010)
Rat hippocampal and dorsal root ganglia	Filopodia	0.001	0.005	0.005	OT	Amin et al. (2013)
Rat hippocampal and dorsal root ganglia	Lamellipodia	0.001	0.02	0.02	OT	Amin et al. (2013)
*Aplysia Californica* bag cell neurons	Lamellipodia	0.0001	0.1		OT	Mejean et al. (2013)
Spinal commissural neuron	Growth cone		0.063		OT	Moore et al. (2009)
Spinal commissural neuron	Growth cone	0.002	0.037	0.009	TFM	Moore et al. (2009)
NG108-15	Axon			0.602	TFM	Betz et al. (2011)
Chick sensory neurons	Axon	0.4	4.8	1.3	MN	O’Toole et al. (2015)
*Drosophila* motor neurons *in vivo*	Axon	1	13	7	MEMS	Rajagopalan et al. (2010)
NG108-15	Growth cone		0.15	0.102	AFM	Fuhs et al. (2013)
Mouse retinal ganglion cell	Growth cone		0.17	0.115	AFM	Fuhs et al. (2013)
*Aplysia Californica* bag cell neurons	Growth cone	82	158	120	AFM	Athamneh et al. (in press)
*Aplysia Californica* bag cell neurons	Growth cone	2.5	92.2	20.3	MN	Athamneh et al. (in press)

*AFM, atomic force microscopy; MEMS, microelectromechanical system-based force sensors; MN, microneedle; OT, optical tweezers; TFM, traction force microscopy*.

Quantitative studies of mechanical forces in axons and growth cones have yielded significant findings about the critical role of force in growth cone dynamics and axonal elongation. However, many important questions remain unanswered, and apparent discrepancies in the literature, particularly related to the effect of substrate stiffness on neurite outgrowth, need to be addressed. Although the details of the mechanism by which growth cones sense and responds to substrate stiffness are still unclear, it appears to be a highly coordinated mechanism involving a combination of biochemical and biomechanical processes spanning multiple length scales. Understanding the details of this mechanism will undoubtedly require and stimulate more research involving quantitative force measurements.

## Conflict of Interest Statement

The authors declare that the research was conducted in the absence of any commercial or financial relationships that could be construed as a potential conflict of interest.
